# Early Precise Prediction and Severe Risk Stratification of Intrahepatic Cholestasis of Pregnancy: Advances and Future Directions in Clinical Translation from Traditional Models to Artificial Intelligence and Multi-Omics Technologies

**DOI:** 10.3390/jcm15155887

**Published:** 2026-07-28

**Authors:** Wenting Xu, Minghe Wang, Xiang Li, Yiqing Li, Shanping Wang, Yan Sun

**Affiliations:** 1 Department of Gastroenterology, Department of Obstetrics and Gynecology, Guangdong Provincial Key Laboratory of Major Obstetric Diseases, Guangdong Provincial Clinical Research Center for Obstetrics and Gynecology, Guangdong-Hong Kong-Macao Greater Bay Area Higher Education Joint Laboratory of Maternal-Fetal Medicine, The Third Affliated Hospital, Guangzhou Medical University, Guangzhou 510150, China; 2024360059@stu.gzhmu.edu.cn (W.X.);; 2 Department of Respiratory and Critical Care Medicine, Department of Obstetrics and Gynecology, Guangdong Provincial Key Laboratory of Major Obstetric Diseases, Guangdong Provincial Clinical Research Center for Obstetrics and Gynecology, Guangdong-Hong Kong-Macao Greater Bay Area Higher Education Joint Laboratory of Maternal-Fetal Medicine, The Third Affliated Hospital, Guangzhou Medical University, Guangzhou 510150, China

**Keywords:** intrahepatic cholestasis of pregnancy, prediction model, artificial intelligence, multi-omics technologies, severe ICP

## Abstract

Intrahepatic cholestasis of pregnancy (ICP) is a liver disorder unique to pregnancy, closely associated with severe adverse maternal and fetal outcomes such as preterm birth and intrauterine fetal death. Its pathogenesis involves a complex interplay of genetic, hormonal, metabolic, and environmental factors, with a higher risk observed in southern China and among individuals co-infected with hepatitis B virus. Substantial evidence demonstrates a significant dose–response relationship between serum total bile acid (TBA) levels and perinatal outcomes, with TBA ≥ 40 μmol/L commonly used as a criterion for severe ICP. However, most existing prediction models are based on single-center, retrospective studies with small sample sizes and insufficient external validation, limiting their clinical generalizability. In recent years, nomograms and machine learning methods have demonstrated advantages in the early prediction and risk stratification of ICP. Deep learning models and multi-omics integration strategies have further enhanced predictive accuracy. Nevertheless, challenges remain regarding model interpretability, data standardization, and cross-population applicability. Future research should leverage large-scale, multi-center prospective cohorts, integrating multi-omics technologies, including genomics, metabolomics, gut microbiome profiling with artificial intelligence to develop clinically actionable and interpretable risk prediction tools. Integrating these tools into electronic health record systems and mobile platforms may facilitate the early identification and individualized management of ICP, ultimately improving maternal and neonatal outcomes.

## 1. Introduction

Intrahepatic cholestasis of pregnancy (ICP) is a pregnancy-specific liver disorder characterized by pruritus and elevated serum bile acid, typically occurring in the mid-to-late trimester. Although symptoms usually resolve postpartum, ICP is strongly associated with serious adverse maternal and fetal outcomes. Early prediction and precise risk stratification remains a pressing clinical need. The global incidence of ICP varies widely, ranging from approximately 0.1% in Northern Europe to as high as 15% in South America. While a recent systematic review estimated a pooled global incidence of 2.9%, this average masks substantial regional heterogeneity [1]. In China, estimates range from 0.3% to 6.0%, with higher rates reported in southern regions such as Guangdong and Chongqing [2,3,4].

Although most women have a favorable maternal course, ICP significantly increases the risk of preterm birth, premature rupture of membranes, fetal distress, and intrauterine fetal death [5]. Previous studies have confirmed a strong correlation between ICP severity and total bile acid (TBA) concentration. When TBA is greater than 40 μmol/L, the risks of preterm birth and perinatal mortality increase significantly. When TBA exceeds 100 μmol/L, the risk of intrauterine fetal death rises markedly [4,6]. Accordingly, both domestic and international guidelines use TBA levels as a key basis for disease stratification and clinical guide management, emphasizing intensified monitoring and timely intervention for women with severe ICP [5].

Despite these guidelines, current clinical practice faces two major dilemmas: firstly, the lack of effective predictive tools to identify high-risk women in the first or second trimester; and secondly, the absence of prospective models capable of identifying the risk of progression to severe ICP before it occurs. Recent studies have suggested potential risk factors for ICP including hepatitis B virus infection, elevated liver enzymes, gestational diabetes, and multifetal gestation, but much of evidence comes from small-sample, single-center studies, limiting generalizability and clinical utility for prediction [2,7,8]. At the same time, advances in precision medicine and artificial intelligence (AI) have prompted efforts to improve early prediction and risk stratification for ICP using multivariable models, machine learning, and multi-omics integration. By integrating clinical features with laboratory parameters and emerging data types, such as metabolomic, genomic, and gut microbiome, these methods may facilitate the discovery of novel predictive biomarkers and support more targeted management of high-risk pregnancies [9].

Compared with previous reviews, the present manuscript offers several distinctive contributions. First, while Wang et al. [10] comprehensively summarized omics findings in ICP, our review specifically emphasizes the integration of multi-omics discoveries with AI-based prediction models for severe-risk stratification. Second, we provide a structured comparative analysis of contemporary machine learning approaches (including CatBoost, RNN, and LASSO-based models) and critically appraise their methodological limitations—an aspect not systematically addressed in prior reviews. Third, we present a comparative synthesis of international guidelines (SMFM, RCOG, JOGC) to inform clinical decision-making. Collectively, this review aims to bridge the gap between emerging technologies and clinical practice by offering a clinically oriented framework for early prediction and risk stratification of severe ICP.

To interpret these findings, it is essential to review the epidemiological characteristics and pathogenesis of ICP, which informs both the identification of high-risk populations and the biological rationale for candidate predictors.

## 2. Materials and Methods

This narrative review was conducted by systematically searching the PubMed(National Library of Medicine, Bethesda, MD, USA), Web of Science (Clarivate, Philadelphia, PA, USA), and Google Scholar (Google, Mountain View, CA, USA) databases. Relevant literature published between 2020 and 2025 was considered, with no language restrictions applied. The search strategy combined terms related to ICP (“intrahepatic cholestasis of pregnancy” OR “ICP”) with each of the following themes: prediction models (“prediction model” OR “nomogram” OR “risk score”), artificial intelligence (“machine learning” OR “deep learning” OR “neural network”), multi-omics (“genomics” OR “metabolomics” OR “microbiome” OR “biomarker”), and epidemiology (“incidence” OR “prevalence” OR “risk factor”). Additional relevant studies were identified by screening the reference lists of retrieved articles. Inclusion criteria were: (1) studies on ICP prediction, risk factors, or severity stratification; (2) machine learning or multi-omics studies related to ICP; and (3) systematic reviews, meta-analyses, and clinical guidelines.

## 3. Epidemiology and Pathogenesis of ICP

### 3.1. Epidemiological Characteristics

A recent global systematic review estimated the overall incidence of intrahepatic cholestasis of pregnancy (ICP) at approximately 2.9% (95% CI, 2.5–3.3%), with marked geographical variation across regions [1]. In China, epidemiological studies further demonstrate a clear regional gradient, with a higher incidence reported in southwestern and southern provinces such as Chongqing and Guangdong [1].

Such regional differences suggest that ICP occurrence is influenced not only by genetic susceptibility but also contextual factors, including environmental exposures, viral infections, and nutrition status. Population-specific studies show that several maternal characteristics have been associated with increased risk, including multiple pregnancies, advanced maternal age, multiparity, and a history of contraceptive pill-related cholestasis [11]. Furthermore, large-scale studies in China indicate that hepatitis B virus (HBV) infection is a significant risk factor, with HBsAg-positive pregnant women having a markedly increased risk of developing ICP [8]. These findings suggest that pregnant women in southern China and those with HBV infection may benefit from enhanced clinical screening and surveillance.

**Figure 1:**
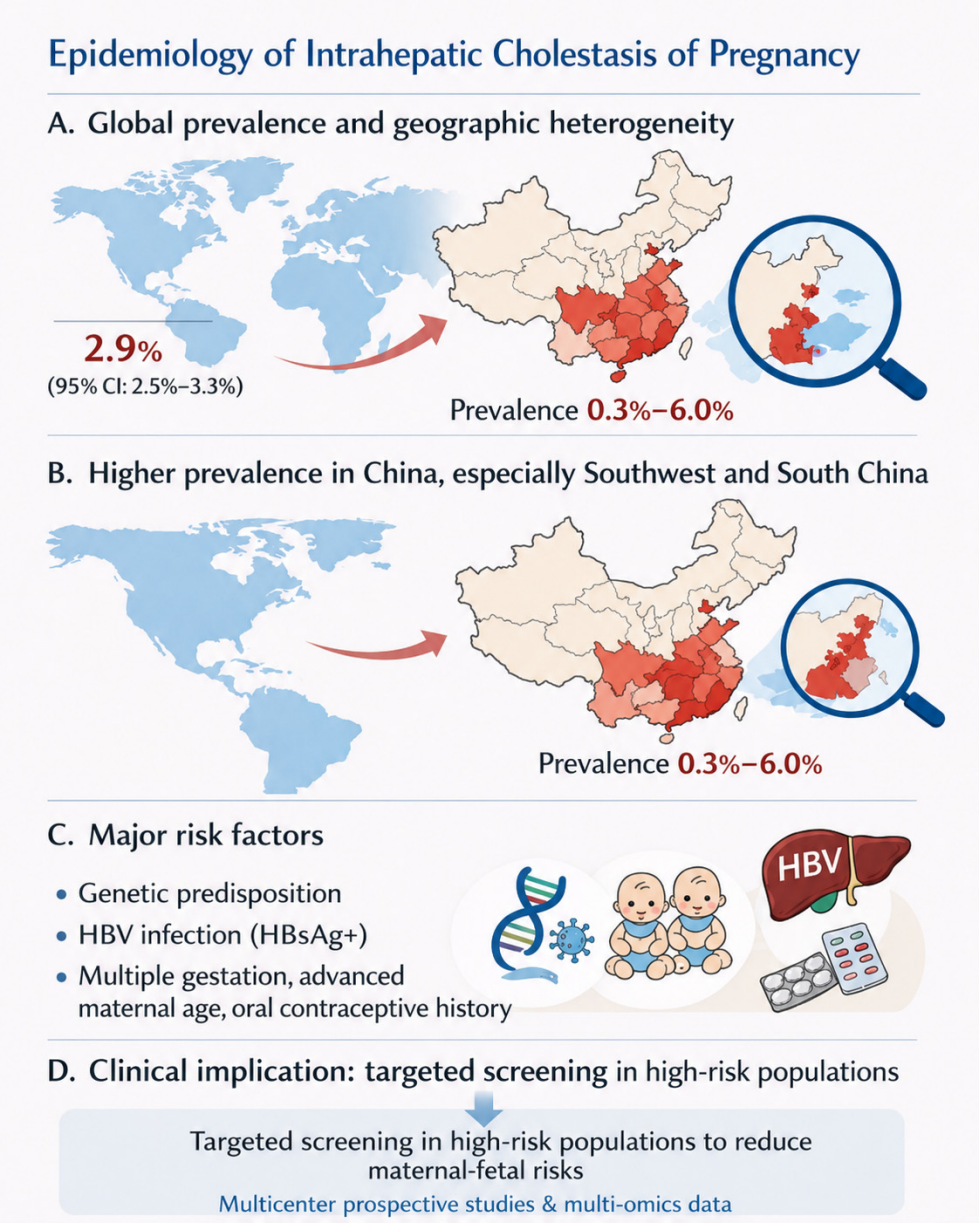
Epidemiological characteristics of intrahepatic cholestasis of pregnancy (ICP). The figure illustrates geographic variation, major risk factors, and implications for targeted screening.

Together, these epidemiological differences and etiological signals underscore the multifactorial nature of ICP and highlight the importance of identifying high-risk populations for early screening and individualized management (Figure 1).

### 3.2. Pathogenesis

The etiology of ICP is complex, widely considered to result from the combined effects of genetic susceptibility, hormonal changes, and environmental or metabolic factors. In the mid-to-late trimester, significantly elevated estrogen and progesterone levels can inhibit the function of hepatocyte bile acid efflux pumps (BSEP/ABCB11) and multidrug resistance-associated protein 2 (MRP2) [12]. This impairment limits bile acid excretion, thereby promotes bile acid accumulation, and contributes to clinical manifestations such as pruritus [12].

In recent years, multi-omics technologies have been increasingly applied to ICP pathogenesis research, clarifying genetic susceptibility and metabolic abnormalities. For example, genomic studies have found that mutations in bile acid transporter-related genes, particularly ABCB4 and ABCB11, are closely associated with ICP risk and severity, particularly more common in severe or early-onset cases [13]. Metabolomic studies further revealed characteristic disturbances in the bile acid composition, where elevated levels of glycine- and taurine-conjugated bile acids are highly correlated with preterm birth and adverse fetal outcomes [10]. Collectively, these findings suggest that multi-omics approaches can not only help elucidate the disease biology but also may identify novel candidate biomarkers and intervention targets, which may be particularly valuable in high-incidence populations such as those in southern China.

In summary, the pathogenesis of ICP reflects gestational hormonal effects on bile acid transport together with genetic mutations, metabolomic abnormalities, with inflammatory pathways likely contributing. Future research should integrate multi-omics data with AI-based models to better define disease mechanisms and promote the application of pathogenesis-related markers into clinical tools for early prediction and risk stratification.

**Figure 2:**
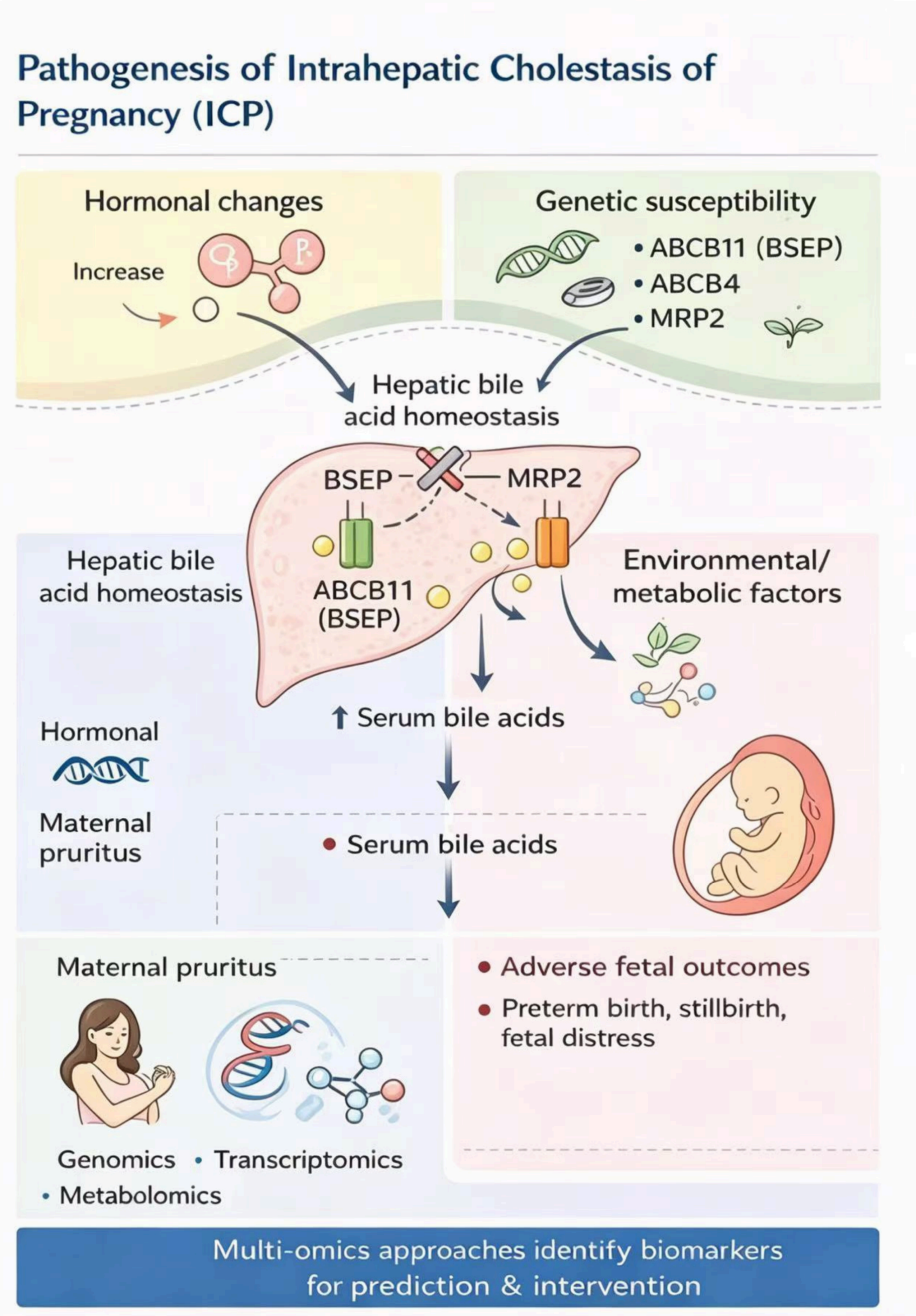
Proposed mechanisms involved in ICP pathogenesis. Hormonal, genetic, and metabolic alterations converge to disrupt bile acid homeostasis and contribute to maternal–fetal complications. The upward arrow (↑) denotes increased serum bile acid levels.

These findings indicate that ICP results from a complex interaction between hormonal regulation, genetic susceptibility, metabolic disturbance, and bile acid transport dysfunction. The proposed mechanisms are summarized in Figure 2.

## 4. Maternal and Fetal Outcomes and Clinical Significance of ICP

ICP affects maternal health and significantly increases the risk of adverse perinatal outcomes. For the mother, ICP is often associated with higher rates of cesarean section, postpartum hemorrhage, and an increased risk of other pregnancy complications. Some women may also experience liver dysfunction or fat-soluble vitamin malabsorption [11].

For the fetus, intrahepatic cholestasis of pregnancy is consistently associated with an increased risk of adverse perinatal outcomes, including preterm birth, meconium-stained amniotic fluid, and fetal distress. Importantly, accumulating evidence supports a clear dose–response relationship between maternal serum total bile acid (TBA) levels and fetal risk, with progressively higher bile acid concentrations conferring greater perinatal vulnerability [14,15].

Accordingly, clinical guidelines recommend using bile acid levels as a crucial basis for disease stratification and clinical management. Particularly, for women with severe ICP (TBA ≥ 40 μmol/L), intensified antenatal monitoring and surveillance are recommended, with early delivery considered when necessary are advised to reduce perinatal risks [5]. Therefore, accurately identifying high-risk patients, optimizing delivery timing, and developing individualized management plans are of great clinical importance for improving maternal and neonatal outcomes. A subsequent 2024 systematic review further confirmed the association of ICP with adverse outcomes such as preterm birth, neonatal intensive care unit (NICU) admission, and preeclampsia, reinforcing the clinical necessity for early identification and risk stratification in this population [16].

## 5. Current Status of Early Prediction Research for ICP

In recent years, several studies have attempted to develop ICP prediction models in the first or second trimester to enable earlier identification of high-risk populations. Most current models, however, remain at the stage of single-center retrospective analysis, lacking external validation, which limits their clinical generalizability. Traditional research has largely focused on clinical risk factors such as multiple pregnancy, advanced maternal age, multiparity, and abnormal liver function indices, but the predictive value suggested by these single factors is limited, and often insufficient for clinical decision-making [17]. To address this limitation, multivariable-based nomogram models have been increasingly used. For example, Zhang Yong et al., reported that a model combining pregnancy characteristics and laboratory indicators could effectively estimate ICP risk in a Chinese cohort [18].

Beyond traditional modeling methods, artificial intelligence (AI) algorithms have recently been introduced into the field of obstetric disease prediction. Machine learning and deep learning models can integrate complex, multi-dimensional data to improve predictive accuracy. One study constructed a machine learning model incorporating gestational age, TBA levels, and clinical features, showing good discrimination and calibration on internal validation [19]. Additionally, AI methods such as deep neural networks (DNNs) and recurrent neural networks (RNNs) have also demonstrated promising performance in small-sample cohorts, though their robustness and generalizability remain uncertain [20]. Overall, ICP prediction research is evolving from single-factor analysis towards multifactor comprehensive modeling, with growing use of AI-based methods. Future research should prioritize large, multi-center prospective cohorts, follow TRIPOD standards, and strengthen clinical utility evaluation (e.g., decision curve analysis) to promote the implementation of prediction tools in prenatal follow-up and individualized delivery decision-making.

## 6. Research on Severe Risk Stratification for ICP

### 6.1. Definition and Clinical Significance of Severe ICP

According to current domestic and international guidelines, disease severity in intrahepatic cholestasis of pregnancy is primarily stratified based on maternal serum total bile acid (TBA) levels, with a threshold of ≥40 μmol/L widely adopted to define severe ICP [5]. This cutoff is supported by robust evidence from large-scale cohort studies and meta-analyses, demonstrating a significantly increased frequency of adverse maternal and fetal outcomes, including preterm birth, fetal distress, and perinatal mortality above this level [14,15]. Notably, a population-based study involving more than 15,000 pregnancies further confirmed a graded, dose–response relationship between bile acid concentrations and adverse outcomes, providing an evidence-based framework for risk-oriented clinical stratification [4].

### 6.2. Related Risk Factors

Severe ICP is often accompanied by markedly elevated liver enzyme levels and more typical clinical symptoms. However, most existing evidence comes from univariate analyses, and few findings have been confirmed in multivariable models; predictors of progression to severe disease remains uncertain. Studies indicate that abnormal liver function indicators, elevated serum alanine aminotransferase (ALT) and aspartate aminotransferase (AST) are more common reported in women with severe ICP, suggesting a correlation between the degree of hepatocyte injury and disease severity [11]. Regarding clinical symptoms, pruritus, particularly palmar and plantar pruritus, as well as gastrointestinal discomfort such as nausea and vomiting, are more prominent in severe cases [21]. Furthermore, severe ICP may present earlier in gestation, and this earlier onset is positively correlated with an increased risk of preterm birth, a pattern validated in multiple studies [4].

Recently, multi-omics approaches have provided additional biological insights into the severity stratification of intrahepatic cholestasis of pregnancy. Elevated inflammatory cytokines, such as interleukin-6 (IL-6) and tumor necrosis factor-α (TNF-α), have been consistently observed in women with severe ICP, supporting the involvement of inflammatory pathways in disease progression. In parallel, metabolomic profiling has identified increased levels of glycine- and taurine-conjugated bile acids as potential biomarkers associated with disease severity [7,9].

### 6.3. Clinical Management Challenges and Comparison of International Guidelines

Current evidence regarding risk stratification and clinical management of severe intrahepatic cholestasis of pregnancy (ICP) remains limited. Although maternal serum total bile acid (TBA) concentration is widely recognized as the most important indicator for assessing disease severity and fetal risk, the optimal thresholds for diagnosis, severity classification, and clinical intervention remain under debate. Most available studies have focused on identifying individual clinical or biochemical risk factors, while relatively few have developed and validated comprehensive prediction models incorporating multiple parameters [19]. In addition, existing evidence is often derived from small sample sizes, single-center cohorts, retrospective designs, and heterogeneous definitions of disease severity, limiting the generalizability of findings and their application in routine clinical practice.

In addition to limitations in risk prediction, differences among international guidelines highlight the complexity of clinical decision-making in ICP management. Currently, recommendations from the Society for Maternal-Fetal Medicine (SMFM), the Royal College of Obstetricians and Gynaecologists (RCOG), and the Society of Obstetricians and Gynaecologists of Canada (JOGC) represent the major international references for ICP diagnosis and management. Although these guidelines consistently emphasize the importance of maternal serum bile acid concentrations, variations remain regarding diagnostic thresholds, severity classification, timing of delivery, and fetal surveillance strategies.

Regarding diagnosis, all three guidelines recognize TBA measurement as the cornerstone of ICP assessment; however, the recommended thresholds are not entirely consistent. SMFM considers a TBA concentration of ≥10 μmol/L as supportive of the diagnosis of ICP, whereas RCOG places greater emphasis on risk stratification according to peak bile acid concentrations, classifying ICP as mild (19–39 μmol/L), moderate (40–99 μmol/L), or severe (≥100 μmol/L). The JOGC guideline also highlights the clinical importance of bile acid measurement but acknowledges that diagnostic interpretation may be influenced by differences in laboratory methods, assay performance, and population characteristics. These variations indicate that a single universal diagnostic threshold may not fully capture the heterogeneous clinical spectrum of ICP.

Recommendations regarding timing of delivery also differ among guidelines. SMFM recommends delivery between 36 0/7 and 39 0/7 weeks of gestation for patients with ICP, with consideration of earlier delivery for patients with markedly elevated bile acid levels, particularly those with TBA concentrations ≥ 100 μmol/L. RCOG adopts a more individualized approach based on peak bile acid concentrations and associated fetal risk, recommending planned birth at 35–36 weeks for women with severe ICP (TBA ≥ 100 μmol/L), while later delivery may be considered for patients with lower bile acid levels and no additional risk factors. JOGC similarly supports individualized management, integrating bile acid levels, gestational age, maternal symptoms, and fetal conditions rather than applying a fixed delivery threshold to all patients.

Fetal surveillance represents another area of ongoing debate among guidelines. Although antenatal testing is frequently used in clinical practice, evidence supporting its effectiveness in preventing stillbirth remains limited, as fetal demise associated with ICP may occur unpredictably and may not always be preceded by abnormal surveillance findings. SMFM suggests antenatal surveillance when gestational age is sufficient that abnormal results would lead to delivery. In contrast, RCOG emphasizes that routine fetal surveillance has uncertain predictive value and recommends focusing primarily on maternal bile acid monitoring and appropriate timing of delivery. JOGC also recognizes the lack of robust evidence supporting a specific surveillance protocol and recommends individualized assessment based on maternal and fetal risk factors.

Overall, current international guidelines demonstrate substantial agreement regarding the central role of bile acid concentrations in ICP management but differ in their interpretation of thresholds and implementation strategies. These discrepancies largely reflect the limited availability of high-quality prospective evidence and the complex relationship between bile acid levels and fetal outcomes. Future studies should prioritize large multicenter prospective cohorts, standardized definitions of disease severity, and validated prediction models integrating clinical parameters with emerging biomarkers. Such efforts may improve risk stratification and facilitate more personalized management strategies for women with ICP.

## 7. Application of Prediction Models and Novel Methods

Artificial intelligence (AI)-based technology has rapid development in risk prediction in ICP [18]. In a Chinese retrospective study of 798 pregnant women, key risk factors including total bile acid, gamma-glutamyl transferase, multiple pregnancy, and hematocrit were screened via LASSO regression, and models were constructed using various machine learning algorithms. Results showed that the CatBoost model achieved strong performance with an AUC of 0.961 and an accuracy exceeding 90%, and outperformed traditional logistic regression in distinguishing healthy pregnant women, mild ICP, and severe ICP [18]. Another study from the Women’s Hospital, Zhejiang University School of Medicine, evaluated machine learning and deep learning models in 1092 pregnant women, using 62 clinical and laboratory variables collected across pregnancy. Results indicated that the Recurrent Neural Network (RNN) performed best when integrating cross-trimester data, with a sensitivity of 97.6%, specificity of 82.1%, and overall accuracy of 90.5%, supporting the value of deep learning for modeling longitudinal changes in pregnancy indicators. Additionally, a case–control study indicated that the Systemic Inflammation Response Index (SIRI) holds significant value in the diagnosis and severity stratification of ICP, effectively distinguishing between mild and severe patients [22].

Although these studies collectively demonstrate the potential of machine learning and deep learning models in ICP prediction, a structured comparison is needed to identify gaps and guide future efforts (Table 1).

The structured comparison in Table 1 underscores a critical gap: nearly all existing models are derived from single-centre retrospective data without external validation, limiting their generalisability. Beyond these design limitations, additional challenges persist, including the ‘black-box’ nature of deep learning models, which undermines clinical interpretability, and the absence of unified standards for predictor selection and outcome definition. Addressing these issues requires future studies to prioritise prospective, multi-centre designs, rigorous external validation, and the integration of explainable AI techniques to bridge the gap between research and clinical practice.

Despite these encouraging results, the reported performance metrics warrant careful scrutiny. Ren et al. reported an AUC of 0.961 for the CatBoost model in a cohort of 798 women; however, this high discrimination likely reflects overfitting, given the relatively modest sample size for a complex ensemble algorithm, and the complete absence of external validation precludes any assessment of generalisability. Similarly, He et al. achieved a sensitivity of 97.6% and specificity of 82.1% using a recurrent neural network (RNN) in 1092 women from a single centre—yet such an extremely high sensitivity may be inflated by potential data leakage across trimesters or imbalanced class distribution, and the model’s architectural complexity further exacerbates the risk of overfitting to local practice patterns. The nomogram proposed by Xie et al. lacks reported AUC or calibration metrics, rendering its true discriminatory ability unverifiable. The case–control design adopted by Ipek et al., which relies on a single biomarker (SIRI), is inherently prone to spectrum bias and may not reflect real-world diagnostic performance.

### 7.1. Methodological Considerations for AI-Based Prediction Models

Beyond the specific limitations of individual studies, several cross-cutting methodological issues warrant careful consideration when evaluating AI-based prediction models for ICP.

#### 7.1.1. Model Explainability and Clinical Interpretability

A fundamental barrier to clinical adoption of AI models is their inherent “black-box” nature. While complex algorithms such as deep neural networks and ensemble methods often achieve superior predictive performance, they do not readily reveal why a particular prediction was made—a requirement for clinician trust and informed decision-making. In obstetric settings, where decisions directly affect both maternal and fetal wellbeing, this lack of transparency is particularly problematic.

To address this, explainable artificial intelligence (XAI) techniques have been developed to provide post hoc interpretability. Two widely adopted methods are SHAP (SHapley Additive exPlanations) and LIME (Local Interpretable Model-agnostic Explanations). SHAP quantifies the contribution of each feature to a model’s prediction based on cooperative game theory, providing both global (overall feature importance) and local (individual patient-level) explanations. LIME, by contrast, approximates the model locally with an interpretable surrogate model to explain individual predictions. These methods have been increasingly applied in obstetric prediction studies—for example, in predicting birthweight, preterm birth, and cesarean section following induction of labour—demonstrating their feasibility in maternal–fetal medicine.

Importantly, interpretability is not binary but exists on a spectrum. Inherently interpretable models (e.g., logistic regression, decision trees with limited depth) trade some predictive flexibility for transparency, whereas post hoc XAI methods attempt to retrofit explainability onto complex “black-box” models. For ICP prediction, a pragmatic approach might involve comparing both types of models: simpler, interpretable models for initial risk stratification, and more complex models with XAI explanations for high-risk cases where additional predictive accuracy is clinically warranted. Regardless of the approach, model explanations must be aligned with clinical reasoning to be useful at the bedside. Simply providing SHAP summary plots without contextualising them within obstetric pathophysiology may not translate into actionable clinical insights.

#### 7.1.2. Model Calibration

While discrimination (typically measured by AUC) indicates how well a model distinguishes between outcomes, calibration—the agreement between predicted probabilities and observed event rates—is equally critical for clinical decision-making. A model with excellent AUC but poor calibration may systematically overestimate or underestimate risk, leading to inappropriate clinical management.

Calibration is typically assessed using calibration plots, which compare predicted probabilities (x-axis) against observed frequencies (y-axis); perfect calibration is represented by a 45-degree diagonal line. The Hosmer–Lemeshow test provides a statistical measure of calibration, with *p* > 0.05 generally indicating good calibration. The Brier score quantifies the mean squared difference between predicted probabilities and actual outcomes, with lower values indicating better calibration. Importantly, calibration should be assessed both overall and across relevant subgroups (e.g., by gestational age, parity, or geographic region) to detect potential performance disparities.

Calibration is particularly relevant to ICP prediction because clinical decisions—such as the timing of delivery or the frequency of fetal monitoring—depend not only on whether a patient is classified as high-risk, but on the precise probability of adverse outcomes. Overestimating risk may lead to unnecessary iatrogenic preterm delivery, while underestimating risk may delay necessary intervention. Notably, none of the ICP prediction models reviewed in this manuscript reported calibration metrics, representing a significant gap in methodological rigour.

#### 7.1.3. Algorithmic Bias and Generalisability

AI models are inherently susceptible to bias, which in obstetric contexts is particularly concerning given historical under-representation of pregnant women in clinical research and substantial inter-population variations in healthcare access, environmental exposures, and genetic backgrounds.

For ICP prediction, several sources of bias are especially relevant. Sampling bias is almost universal among current studies: all models reviewed were developed from single-centre or regionally restricted cohorts, which may not capture the full diversity of disease presentation—particularly relevant for a condition with marked geographic heterogeneity, where southern China and South America show much higher incidences than Northern Europe. Measurement bias may arise when laboratory assays (e.g., TBA quantification) or clinical definitions (e.g., diagnostic criteria for ICP) differ between institutions, compromising cross-study comparability. Label bias is evident in inconsistent outcome definitions across studies—for instance, some define severe ICP as TBA ≥40 μmol/L while others use higher thresholds, leading to models trained on fundamentally different endpoints. Additionally, deployment context bias refers to the mismatch between the original training population and the target population in real-world clinical settings; a model developed in an urban tertiary hospital may not perform adequately in a rural community with different HBV prevalence, nutritional status, or healthcare-seeking behaviours.

Beyond these technical biases, there is a broader concern that AI models may inadvertently amplify existing maternal health disparities. If a model performs well predominantly in the population from which its training data were drawn, but less well in under-represented groups, its deployment could systematically disadvantage those patients. For example, a model trained on data from urban centres in Guangdong may not generalise to populations in western China where ICP risk factors and baseline characteristics differ. Transparent reporting of model performance across relevant subgroups—by region, parity, gestational age, and co-morbidities—is therefore essential to assess fairness and generalisability before clinical deployment.

Mitigating these biases requires deliberate efforts at multiple stages: ensuring that training cohorts are as diverse as possible, conducting rigorous subgroup performance analyses, and explicitly stating the populations to which a model can be safely applied. Until such steps are taken, reported performance metrics should be viewed as setting-specific rather than universally applicable.

#### 7.1.4. Barriers to Clinical Implementation

Even when AI models demonstrate satisfactory performance in research settings, translating them into routine clinical practice faces multiple barriers.

Methodological barriers include the lack of prospective validation studies and the absence of clinical impact trials demonstrating that model-guided care improves patient outcomes. Most current models remain at the retrospective, single-centre stage, and their performance in real-world, prospective settings is unknown. The TRIPOD+AI (Transparent Reporting of a Multivariable Prediction Model for Individual Prognosis or Diagnosis—Artificial Intelligence) guidelines provide a reporting framework for AI prediction models, but adherence in obstetric studies remains low.

Technical barriers include data privacy concerns, the computational complexity of deploying deep learning models in clinical information systems, and the lack of standardised data formats across institutions. Integration with electronic health record (EHR) systems is particularly challenging, as it requires seamless data extraction, real-time risk calculation, and user-friendly presentation of results within existing clinical workflows.

Human and organisational barriers are equally significant. Clinicians may be reluctant to trust “black-box” recommendations without clear explanations, and AI literacy among perinatal healthcare providers remains limited. Furthermore, the absence of clear regulatory frameworks for AI-based clinical decision support systems creates uncertainty around accountability and liability.

Practical strategies to overcome these barriers include: developing lightweight, mobile-friendly tools that integrate with existing clinical workflows; co-designing models with clinician input to ensure relevance and usability; providing interactive visualisations (e.g., SHAP force plots) that make predictions transparent; and embedding models within clinical decision support systems that generate real-time risk alerts rather than requiring separate user interfaces. Ultimately, successful implementation will require multidisciplinary collaboration among data scientists, clinicians, hospital administrators, and regulatory bodies.

**Figure 3:**
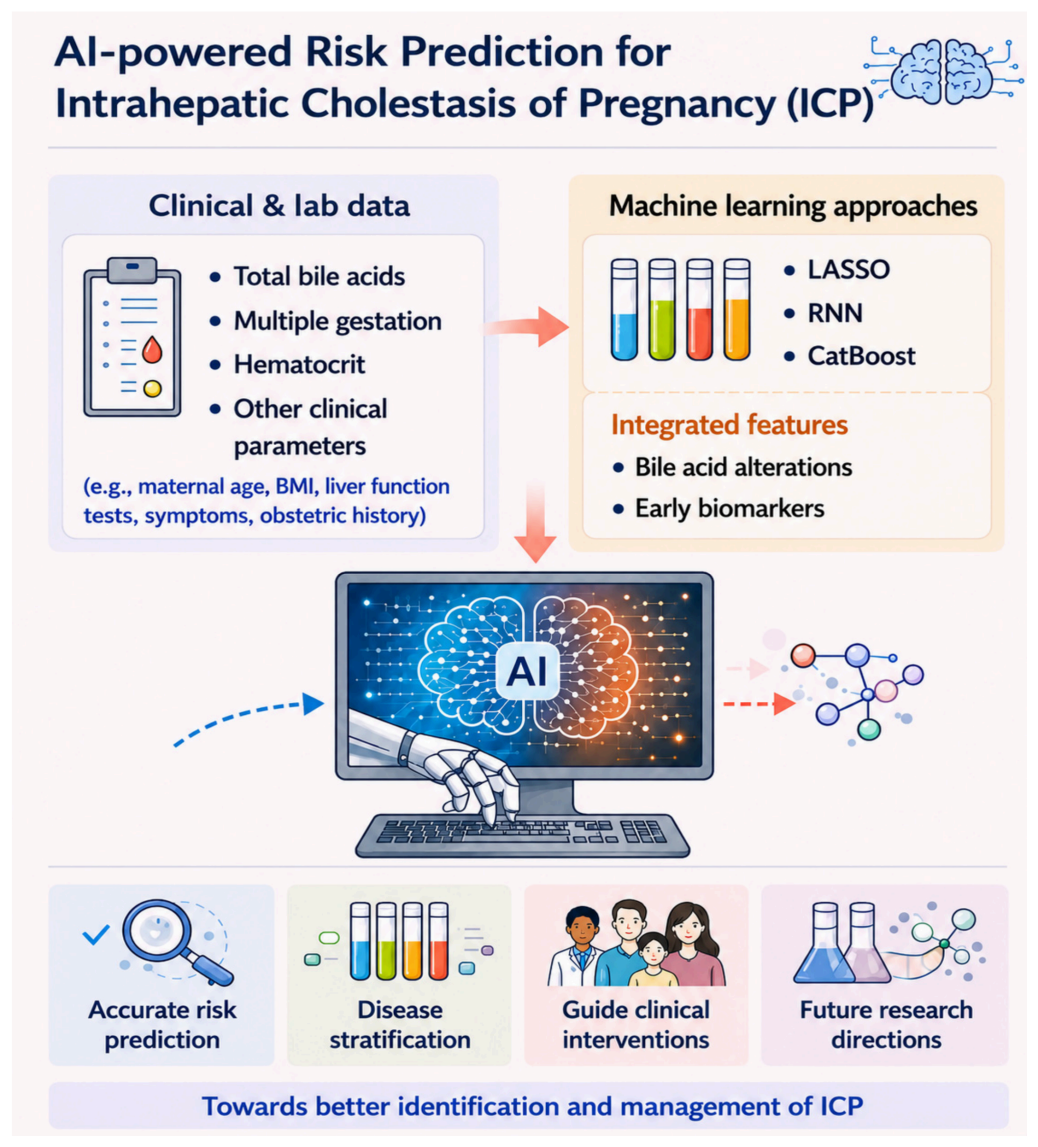
AI-powered risk prediction for ICP. The figure illustrates the workflow of artificial intelligence-based approaches, including clinical data integration, model development, validation, and potential clinical translation for risk prediction and stratification.

Summary. The structured comparison in Table 1 underscores a critical gap: nearly all existing models are derived from single-centre retrospective data without external validation, limiting their generalisability. Beyond these design limitations, additional challenges persist, including the ‘black-box’ nature of deep learning models, which undermines clinical interpretability, and the absence of unified standards for predictor selection and outcome definition. Addressing these issues requires future studies to prioritise prospective, multi-centre designs, rigorous external validation, and the integration of explainable AI techniques to bridge the gap between research and clinical practice (Figure 3).

## 8. Potential of Multi-Omics Technologies in ICP Research

Multi-omics technologies (genomics, transcriptomics, proteomics, metabolomics, and gut microbiomics) offer new perspectives for researching the pathogenesis of intrahepatic cholestasis of pregnancy (ICP). Genomic studies have identified several key gene mutations related to bile acid metabolism, such as ABCB4, ABCB11, and ATP8B1, which are closely associated with the susceptibility and severity of ICP, providing evidence for elucidating the genetic basis of the disease [13]. Metabolomic studies reveal significant abnormalities in the bile acid profile of ICP patients, particularly marked increases in glycine- and taurine-conjugated bile acid levels; these metabolic features are closely linked to preterm birth and adverse fetal outcomes [7]. Further integrated proteomic and metabolomic analyses suggested that bile acid transport–related proteins and selected metabolites may serve as candidate markers for diagnosis and risk stratification, supporting the potential value of multi-omics integration for earlier and more precise prediction [9].

However, despite the identification of numerous candidate biomarkers, none has been successfully implemented in routine clinical practice. Several factors may account for this gap between biomarker discovery and clinical application. First, most current findings are derived from relatively small cohorts, often from single-center or retrospective studies, and require validation in large-scale prospective populations. In addition, differences in ethnicity, geographic background, environmental exposure, and clinical characteristics may affect the reproducibility and generalizability of identified biomarkers. Therefore, whether these molecular signatures can provide consistent predictive value across diverse populations remains uncertain.

Despite these advances, most identified molecular signatures remain at the exploratory stage and have not yet been incorporated into routine clinical practice. The gap between biomarker discovery and clinical implementation reflects several unresolved challenges. First, current evidence is mainly derived from relatively small cohorts, often involving single-center or retrospective studies, and therefore lacks sufficient external validation in large prospective populations. In addition, variations in genetic background, environmental exposure, clinical characteristics, and population composition may influence biomarker performance, limiting the generalizability of findings across different populations. Thus, whether these candidate biomarkers can provide consistent predictive value in diverse clinical settings remains uncertain.

Gut microbiomics research has further expanded the understanding of ICP pathogenesis by revealing the complex interaction between intestinal microorganisms and host bile acid metabolism. Tang et al. demonstrated that increased abundance of *Bacteroides fragilis* in patients with ICP was associated with disrupted bile acid homeostasis, potentially through inhibition of the farnesoid X receptor (FXR) signaling pathway and subsequent alteration of bile acid metabolism [11]. These findings suggest that microbiome-derived signatures may represent promising diagnostic markers or potential therapeutic targets. However, clinical application of microbiome-based biomarkers remains challenging due to substantial interindividual variability and the influence of dietary patterns, geographic background, medication exposure, and sequencing methodologies. Standardized analytical protocols and independent validation studies are therefore required before microbiome signatures can be translated into clinical practice.

**Figure 4:**
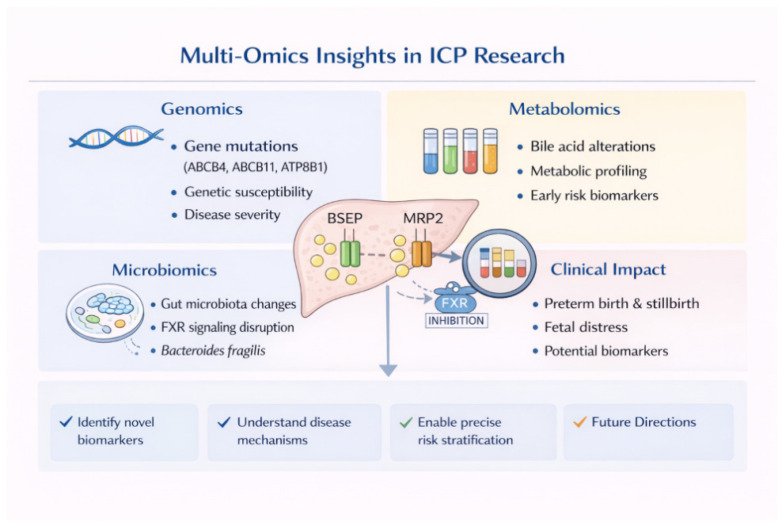
Multi-omics framework for intrahepatic cholestasis of pregnancy (ICP). The figure depicts how genomic, metabolomic, and microbiome-based approaches may contribute to biomarker discovery, mechanistic understanding, and future precision risk stratification.

Beyond biological validation, methodological and practical limitations further restrict the clinical translation of multi-omics discoveries in ICP. Differences in sample collection procedures, sequencing platforms, metabolomic detection methods, and bioinformatic pipelines have resulted in substantial heterogeneity among studies, making cross-study comparison and establishment of standardized assays difficult. Furthermore, the high dimensionality and complexity of multi-omics datasets require advanced computational approaches to generate clinically interpretable, reproducible, and cost-effective predictive tools. Future research should focus on well-designed multicenter prospective cohorts, harmonized analytical workflows, and rigorous external validation of multi-omics-based models. Combining multi-omics information with artificial intelligence approaches may provide an effective strategy to transform molecular discoveries into clinically applicable tools for precision prediction and risk management of ICP (Figure 4).

## 9. Future Directions and Clinical Translation

Although significant progress has been made in recent years regarding the epidemiology, maternal–fetal outcome analysis, and prediction model development for ICP, several limitations remain in current research. Firstly, most studies are retrospective in design, with limited sample sizes and predominantly single-center data, resulting in critically insufficient external validation that limits the generalizability of findings. Secondly, the selection of predictive indicators lacks standardized criteria, and different studies employ varying endpoints (e.g., bile acid thresholds, definitions of preterm birth), leading to poor comparability between models and hindering their widespread adoption [6]. Furthermore, most existing models remain at the research level, have not been integrated into clinical information systems and tool-based applications, limiting their utility in real-world clinical decision-making.

Future research should focus on three key aspects. First, future studies should leverage multi-center prospective cohorts, combining clinical, laboratory, and multi-omics data to develop prediction tools with both accuracy and interpretability. Simultaneously, efforts should be made to integrate these models into electronic health record systems to enable real-time risk alerts, thereby truly implementing precision medicine concepts in perinatal care practice. Second, exploring novel predictive factors by combining multi-omics technologies (genomics, metabolomics, gut microbiome, etc.) with traditional clinical indicators to improve model accuracy and mechanistic explanatory power. Third, strengthening external validation and evaluating cross-population applicability will be essential to promote the development of prediction tools that can be widely adopted across different regions and ethnicities.

The key direction for clinical translation lies in promoting research findings effectively inform serve clinical decision-making. In the future, prediction models should be deeply integrated with electronic health record systems to form intelligent decision support systems capable of generating real-time risk alerts based on patients’ clinical and laboratory data. Concurrently, bedside visualization tools and mobile health applications could be developed to enhance model accessibility and operational ease. To address the “black-box” problem of AI, explainability techniques should be introduced, enabling doctors to understand the factors driving prediction results, thereby enhancing the effectiveness of doctor-patient communication. Moreover, multidisciplinary collaboration will be crucial for optimizing obstetric management pathways based on prediction models. Combined with supportive policies and clinical guideline, these efforts will be essential for truly achieving the routine clinical integration and widespread application of ICP risk prediction tools.

In addition to methodological and technical challenges, the clinical translation of AI-based ICP prediction models faces several practical barriers. First, rigorous cost-effectiveness analyses are largely lacking, and it remains unclear whether the added predictive accuracy of complex AI models translates into net clinical benefit or healthcare savings, particularly in resource-limited settings. Second, the regulatory pathway for AI-based clinical decision support tools remains uncertain in most countries; unlike conventional medical devices or laboratory tests, software-based prediction models are subject to evolving regulatory frameworks (e.g., the FDA’s SaMD framework or the EU AI Act), and the absence of clear approval pathways creates uncertainty for developers and healthcare institutions. Third, clinician acceptance and trust are critical but often overlooked: obstetricians may be reluctant to adopt ‘black-box’ recommendations without intuitive explanations, and AI literacy among perinatal providers varies widely. Finally, feasibility differs substantially across healthcare systems—implementation in high-resource urban tertiary centres with robust EHR infrastructure differs vastly from that in low-resource rural settings, where data availability, internet connectivity, and technical support are limited. Future translation efforts should therefore adopt a ‘fit-for-purpose’ approach, tailoring model complexity and deployment strategies to the specific clinical context and available resources. Regulatory guidance, clinician training, and health economic evaluations should be integrated early in the development pathway to facilitate sustainable implementation.

## 10. Summary and Outlook

Intrahepatic cholestasis of pregnancy represents a clinically important pregnancy-specific liver disorder with substantial heterogeneity in disease presentation and outcomes. Accumulating evidence highlights the necessity of moving beyond single-factor assessment toward integrated prediction and risk stratification strategies.

Recent advances in multivariable modeling, artificial intelligence, and multi-omics technologies have provided new opportunities to improve the early identification of high-risk populations and to better characterize disease progression. However, the translation of these approaches into routine clinical practice remains constrained by issues related to data quality, model interpretability, and generalizability.

Future progress in this field will depend on well-designed multicenter prospective studies and the development of clinically interpretable prediction tools. Strengthening the bridge between methodological innovation and practical clinical application will be essential to enable precision management and ultimately improve maternal and neonatal outcomes in intrahepatic cholestasis of pregnancy.

## Figures and Tables

**Table 1:** Summary of representative prediction models for intrahepatic cholestasis of pregnancy.

	Ren et al. [18]	He et al. [20]	Xie et al. [19]	Ipek et al. [22]
Study design	Retrospective cohort	Retrospective cohort	Retrospective cohort	Case–control
Sample size	798	1092	NR	NR
Population/setting	Single-center, Chinese	Single-center (Zhejiang), Chinese	Single-center, Chinese	Tertiary centre, Turkish
Predictors	TBA, GGT, multiple pregnancy, hematocrit, etc.	62 clinical/lab variables across trimesters	Bile acid levels, maternal characteristics	SIRI
Outcome definition	Mild vs. severe ICP (TBA ≥ 40 μmol/L)	ICP diagnosis (criteria not specified)	Preterm birth in ICP patients	Mild vs. severe ICP
Modeling approach	LASSO + CatBoost	RNN, DNN, etc.	Nomogram (logistic regression)	Logistic regression (implied)
Performance	AUC = 0.961; Acc > 90%	Sens 97.6%, Spec 82.1%, Acc 90.5%	NR	NR
Calibration	Not reported	Not reported	Not reported	Not reported
External validation	None	None	None	None

## Data Availability

As this study is a narrative review based exclusively on the previously published literature, no new primary data were collected or analyzed, and therefore ethical approval and informed consent were not required; all data referred to are available in the cited publications.

## Abbreviations

The following abbreviations are used in this manuscript:

Abbreviation	Full Form
ABCB11	ATP-binding cassette subfamily B member 11
AI	Artificial Intelligence
ALT	Alanine aminotransferase
AST	Aspartate aminotransferase
BSEP	Bile salt export pump
CatBoost	Categorical Boosting
DNN	Deep Neural Network
FXR	Farnesoid X receptor
HBsAg	Hepatitis B surface antigen
HBV	Hepatitis B virus
ICP	Intrahepatic Cholestasis of Pregnancy
IL-6	Interleukin-6
LASSO	Least Absolute Shrinkage and Selection Operator
MRP2	Multidrug resistance-associated protein 2
NICU	Neonatal intensive care unit
RNN	Recurrent Neural Network
SIRI	Systemic Inflammation Response Index
TBA	Total bile acid
TNF-α	Tumor necrosis factor-α
TRIPOD	Transparent Reporting of a Multivariable Prediction Model for Individual Prognosis or Diagnosis
